# Light-triggered enzymatic reactions in nested vesicle reactors

**DOI:** 10.1038/s41467-018-03491-7

**Published:** 2018-03-15

**Authors:** James W. Hindley, Yuval Elani, Catriona M. McGilvery, Simak Ali, Charlotte L. Bevan, Robert V. Law, Oscar Ces

**Affiliations:** 1 Department of Chemistry, Imperial College London, South Kensington, London, SW7 2AZ UK; 2 Institute of Chemical Biology, Imperial College London, South Kensington, London, SW7 2AZ UK; 3 Department of Materials, Imperial College London, Prince Consort Road, London, SW7 2AZ UK; 4 Department of Surgery and Cancer, Imperial Centre for Translational & Experimental Medicine, Imperial College London, Shepherd’s Bush, London, W12 0UQ UK

## Abstract

Cell-sized vesicles have tremendous potential both as miniaturised pL reaction vessels and in bottom-up synthetic biology as chassis for artificial cells. In both these areas the introduction of light-responsive modules affords increased functionality, for example, to initiate enzymatic reactions in the vesicle interior with spatiotemporal control. Here we report a system composed of nested vesicles where the inner compartments act as phototransducers, responding to ultraviolet irradiation through diacetylene polymerisation-induced pore formation to initiate enzymatic reactions. The controlled release and hydrolysis of a fluorogenic β-galactosidase substrate in the external compartment is demonstrated, where the rate of reaction can be modulated by varying ultraviolet exposure time. Such cell-like nested microreactor structures could be utilised in fields from biocatalysis through to drug delivery.

## Abstract (web summary)

Matryoshka doll-like, nested vesicles, each containing a different ingredient to a chemical reaction, can serve as microreactors. Here, the authors developed a system in which mixing of the ingredients can be induced by irradiation with ultraviolet light.

## Introduction

One of the critical steps in the emergence of single celled life was the development of the membrane^1^. Such structures enable the demarcation of space critical for the emergence of self-replicating chemical systems. The development of sub-cellular organelles in eukaryotes has allowed further specialisation of function, from energy generation to biomolecule degradation, which can occur orthogonally to the numerous biochemical processes necessary for maintenance of homoeostasis^2^. Biological cells can be viewed as incredibly complex microreactors, which possess the ability to conduct thousands of biochemical reactions in parallel, whilst simultaneously controlling the selective transport of molecules between compartments and the external environment. Inspired by the structural complexity found in nature, the field of bottom-up synthetic biology has been established which looks to create the ‘minimal cell’^3–5^, to establish cellular functions such as reproduction, metabolism and gene expression^6–10^, and ultimately utilise such organisms for varied applications.

A recent focus in the creation of synthetic cells is to create multi-tiered mimics of cellular architecture. Diverse compartmentalised systems have been fabricated, including vesicle-in-vesicle^11–14^, polymersome-in-polymersome^15^ and more exotic hierarchical proteinosome structures^16^. These systems vary in the size and number of compartments and incorporate varied functions, such as multi-compartment-spanning enzyme cascades^15^ and cargo release properties^16^. Whilst each of the above structures can function as microreactors, lipid vesicles with ultra-low (picolitre-femtolitre) volumes are especially useful due to their physiological compatibility and integration with biological machinery. One feature currently lacking, however, is the incorporation of chemical motifs that allow multi-compartment vesicle systems to respond to optical cues. Incorporation of such motifs enables the creation of structures that combine the increased spatiotemporal control of chemical reactions gained through compartmentalisation with greater system responsiveness obtained via optical control. The use of photo-responsive moieties in multi-compartment structures is in its infancy, with pioneering work focusing on the development of layer-by-layer deposited^17^ and hydrogel polymeric structures^18^, where the inner compartment is destroyed upon optical illumination, facilitating biomolecular reactions and cargo release. More recent work has been undertaken by Bayley and co-workers, where artificial tissue was created capable of responding to light by expressing the peptide-based pore α-hemolysin^19^.

The incorporation of diacetylene functional groups in lipid systems confers non-destructive photo-responsiveness to a membrane^20^. Pore formation is obtained through cross-linking of diacetylene functional groups present in the lipid 1,2-bis(10,12-tricosadiynoyl)-sn-glycero-3-phosphocholine (DC_89_PC). Clustering of DC_89_PC in the bilayer is necessary to obtain high efficiencies of photopolymerisation, where the production of ene-yne conjugates via 1,4 addition occurs under ultraviolet (UV-C, *λ* = 254 nm) irradiation^21^. DC_89_PC-domain cross-linking then results in the creation of pores via the formation of domain boundary defects, akin to the defects generated when single phase lipid systems undergo their gel to fluid transition^22^.

Here we present a lipid-based, cell-sized nested vesicle system (vesicles-in-vesicles) where the inner compartments act as phototransducers, responding to UV irradiation through DC_89_PC polymerisation-induced pore formation. Such systems give high levels of control over the mixing of different encapsulated components of the microreactor. We illustrate this through separation of the enzyme β-galactosidase (β-gal), an exoglycosidase enzyme important for lactose metabolism^23^, and its non-fluorescent fluorogenic substrate fluorescein di-β-D-galactopyranoside (FDG) in different compartments. Enzymatic catalysis is achieved through the application of UV-C radiation, where the substrate diffuses out of the inner compartment and is exposed to the enzyme, generating the fluorescent product fluorescein. By modulating irradiation time, reaction kinetics can be altered as desired, which is suitable for downstream applications of the vesicles including biocatalysis.

## Results

### Controlled catalysis in light-responsive nested vesicles

Four criteria are key in the design of UV-responsive, nested vesicle reactors: (i) long-term membrane stability of all compartments (ii) minimising undesired permeability to ensure content separation (iii) choosing a lipid composition that ensures high DC_89_PC photopolymerisation efficiency for the inner compartments and (iv) high encapsulation efficiency of all components within the formed reactor.

With these four factors in mind, 1,2-dipalmitoyl-sn-glycero-3-phosphocholine: 1,2-bis(10,12-tricosadiynoyl)-sn-glycero-3-phosphocholine: 1,2-distearoyl-sn-glycero-3-phosphoethanolamine-N-[methoxy(polyethylene glycol)-2000 (DPPC:DC_89_PC:DSPE-PEG2000 79.5:20:0.5) vesicles, inspired by work from Yavlovich et al. were chosen for the inner lipid compartment^24^, whilst 1-palmitoyl-2-oleoyl-*sn*-glycero-3-phosphocholine (POPC) was chosen for the external membrane.

**Fig. 1:**
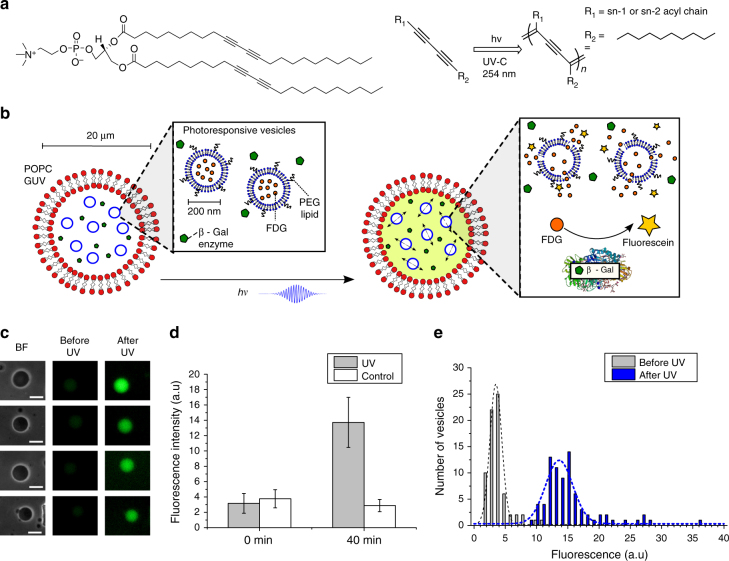
UV-responsive nested vesicles can be created via phase-transfer. **a** Chemical structure of DC_89_PC and the UV-catalysed polymerisation of diacetylene molecules. **b** Cartoon of UV-responsive nested vesicle mechanism. Photo-responsive vesicles and the enzyme β-galactosidase are co-encapsulated within POPC GUVs created via phase-transfer. Upon illumination with UV-C, photopolymerisation of the inner compartment membranes results in FDG release, leading to its catalysis by β-galactosidase forming free fluorescein. **c** Optical microscopy of UV-responsive vesicles before UV irradiation and 30 min after UV irradiation. Scale bar in all images, 25 μm. **d** Comparison of mean fluorescence of UV-responsive nested vesicles before and after 10 min irradiation and 30 min wait. Controls are left for 40 min without application of UV. Error bars represent 1 s.d. (*n* = 71/79 for before/after UV). **e** Histogram of vesicle population in C before and after UV

Emulsion phase-transfer (EPT) is an established method of creating giant unilamellar vesicles (GUVs) with control of membrane asymmetry^7,25–28^. Here, adaptation of the centrifuge-driven EPT method described by Fujii et al is used, creating emulsions containing UV-responsive large unilamellar vesicles (LUVs) extruded to ~200 nm in diameter and β-galactosidase in the aqueous phase, and POPC dissolved in mineral oil^29^. These emulsions are then driven through a lipid-stabilised oil–water interface to create the outer membrane (Fig. 1).

Each nested reactor contains two main components: FDG-loaded light-responsive vesicles and the enzyme β-galactosidase (Fig. 1b). To test the ability of the nested vesicles to respond to a UV-stimulus, vesicles were exposed to 10 min UV irradiation, and then left for a further 30 min to allow significant catalysis to occur. As shown in Fig. 1c, a clear increase in fluorescence intensity, localised to the nested vesicles, can be observed. This increase can be quantitatively measured through image analysis, as shown in Fig. 1d. A statistically significant fluorescence increase was observed (*p* < 0.0001; unpaired t-test, *n* = 71/79 before/after UV) after 40 min for samples exposed to UV. The observed 5-fold increase upon irradiation indicates that UV light can be utilised as a trigger for controlled mixing and subsequent catalysis.

Due to the inclusion of 0.5 mol% DSPE-PEG, negligible fusion should occur between the internal and external membranes. This was confirmed via control experiments where nested vesicles left unexposed resulted in no fluorescence increase over 40 min, indicating the presence of a stable multi-compartment system.

Finally, to better visualise the spread of fluorescence intensities across the vesicle population, a histogram of the irradiated vesicles was generated (Fig. 1e). It can be seen from applying Gaussian fits to the pre- and post-irradiated intensities that UV irradiation results in the creation of a new vesicle population, due to significant β-gal catalytic activity, and, thus, fluorescence increase over time.

### Probing DC_89_PC polymerisation kinetics and FDG cargo release

The encapsulation efficiency of the internal, UV-responsive LUVs was optimised from published compositions by the Puri group (DPPC:DC_89_PC:DSPE-PEG2000 76:20:4)^24^. It has been shown previously that although the presence of PEG stabilises lipid vesicles, it can also result in decreased encapsulation efficiency^30^. The effect of DSPE-PEG2000 on DPPC:DC_89_PC vesicles was therefore investigated through the encapsulation of 0.5 mM calcein in vesicles extruded through a 100 nm filter. By comparing the fluorescence of purified vesicle formulations containing 0–4 mol% DPSE-PEG2000, relative encapsulation efficiencies can be estimated.

As expected, the encapsulation of calcein decreased with increasing PEG, with ~65% drop in encapsulation from 0.5 mol% to 4 mol% DSPE-PEG2000 (Supplementary Fig. 1). Vesicles containing 0 mol% PEG resulted in high levels of aggregation when incubated at 4 °C, attributed to DC_89_PC tubule formation^31^ and so could not be used in further work, whilst all other formulations showed no changes in vesicle size throughout the experiment (Supplementary Fig. 2). As DPPC:DC_89_PC vesicles containing 0.5 mol% DSPE-PEG2000 possessed the desired stability and highest encapsulation efficiency, this composition was chosen for our experiments.

**Fig. 2:**
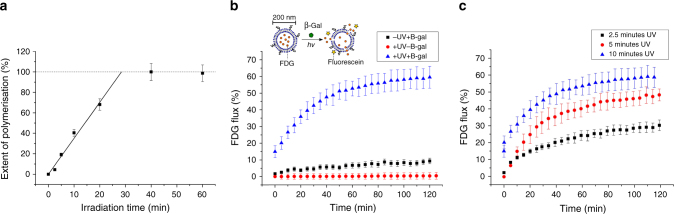
DC_89_PC polymerisation can trigger substrate release. **a** Extent of DC_89_PC polymerisation correlates with UV irradiation time. Extent of polymerisation estimated through normalisation of changes in absorbance at 475 nm, and applying a linear fit to estimate polymerisation saturation (*r* = 0.998). **b** UV-C irradiation can be used to efficiently and quickly release FDG from vesicles containing DC_89_PC. Vesicles irradiated for 10 min, with/without the presence of 5  Uml^−1^ β-galactosidase. **c** FDG release can be modulated by changing irradiation time. β-galactosidase enzyme is present at 5 Uml^−1^ in each case. Error bars represent 1 s.d. (*n* = 3) for each data set

To better understand the kinetics of photopolymerisation, as well as its effect on vesicle stability, UV-vis spectroscopy, dynamic light scattering (DLS) and cryogenic transmission electron microscopy (TEM) were used to characterise UV-responsive vesicles. Ene-yne conjugates created during irradiation show strong absorbance peaks around 480 and 515 nm^20^, and UV-vis spectroscopy can be used to monitor both the kinetics and extent of photopolymerisation in vesicles^32^. The development of this broad peak can be observed, confirming that photopolymerisation is occurring efficiently (Supplementary Fig. 3). Closer observation shows saturation of this peak (centred ~475 nm) occurring ~40 min, as no difference can be observed upon further irradiation. If we assume that saturation of the absorbance peak corresponds with a fully polymerised system, the kinetics of polymerisation can be estimated by normalising against the onset of saturation, as shown in Fig. 2a. Applying a linear fit (*r* = 0.998) and extrapolating to the saturation point allows estimation of a rate of polymerisation of ~3.5%/min. We can also use this linear relationship to estimate the extent of irradiation at a given time; ~50% polymerisation of the system occurs ~14.5 min irradiation, whilst 100% polymerisation occurs ~28 min. Such information is key in understanding the minimum length of irradiation time necessary for triggered release, as well as the potential for modulation of release kinetics.

To confirm that UV irradiation is not causing large-scale disruptions to the light-responsive vesicle membranes that make up the inner compartments of the microreactor, DLS measurements of 200 nm vesicles were performed on vesicles prepared in 20 mM HEPES, 100 mM KCl, pH 7.4, buffer as well as deionised water after 0, 10 and 40 min UV irradiation. As observed in Supplementary Fig. 4, the diameter of the produced vesicles were ~200 nm as expected, and negligible size increases were observed for each sample at both 10 and 40 min (~30% and ~100% photopolymerisation, respectively). Additionally, a low polydispersity index (PDI) was observed for all samples before and after irradiation (PDI < 0.2), indicating that the vesicles are stable and do not undergo membrane lysis or aggregation upon polymerisation. This was further reinforced via cryo-TEM analysis of vesicles irradiated for 10 min (Supplementary Fig. 5). The observed vesicles were shown to be unilamellar, possessing the expected spherical structure and no visible membrane disruption with an average diameter ~220 nm, correlating well with DLS measurements.

Investigations into the rate of FDG release from UV-responsive vesicles were then undertaken. FDG was encapsulated via thin-film rehydration, and unencapsulated FDG removed via size-exclusion chromatography (SEC). SEC resulted in good levels of purification, as shown by the clearly resolved bands for vesicles and free-FDG in the fluorometric elution profile in Supplementary Fig. 6. Rapid release of FDG was obtained through 10 min of UV irradiation (Fig. 2b). Released FDG is hydrolysed by β-gal in external solution (+UV, +β –gal), and 45 min after UV irradiation, ~50% of the encapsulated FDG is released, which is ideal for processes, where large changes in reaction rate are desired. Both UV irradiation and β-galactosidase are necessary for the generation of free fluorescein, as negligible fluorescence increases are observed upon removal of either of these factors. Finally, it was confirmed that FDG release was not due to UV-induced membrane damage through irradiation of FDG-loaded DPPC vesicles. 10 min irradiation of these vesicles in the presence of β-gal resulted in no fluorescence increase over two hours (Supplementary Fig. 7).

Further evaluation of vesicle release properties was undertaken through modulation of UV irradiation time. As the extent of polymerisation is clearly different at 2.5, 5 and 10 min (8.65, 17.6 and 35%, respectively), the ability to modulate release rate is expected. As shown in Fig. 2c, FDG release can be triggered with as little as 2.5 min irradiation, and release rate can be successfully increased two- and four-fold by increasing irradiation time from 2.5 to 5 and 10 min, respectively. Successful optical modulation of the enzymatic reaction rate is critical for the application of these multi-compartment structures. This rate directly influences downstream events, and its modulation illustrates the high level of user control obtainable with this release mechanism.

## Discussion

Engineering light-responsive functionality into vesicle reactors enables the induction and control of chemical and biochemical processes remotely. Such functionality has been utilised in the field of drug delivery, but its application to bottom-up synthetic biology and microreactors remains unfilled. Here, we engineer light-responsive functionality directly into the lipid chassis. This allows nested vesicle structures to be easily adapted to include any enzyme-substrate pair (assuming the substrate can fit through the ~900 Da molecular weight cut off of the generated nanopores^22^).

Although the energy of UV-C radiation prevents full integration of the microreactors with the key nucleic acid-based processes found in biological systems, it can be successfully used to control reaction processes. Compared to temperature and pH, use of optical cues allows greater user control over reaction rates; achieved as simply as turning a laser on and off. Such spatiotemporal control of catalytic reactions is central to the development of 'one-pot' reaction processes involved in biocatalysis^33^.

Multi-compartment systems have already shown great promise in enabling bio-orthogonal processes to occur^34^, and the multi-compartment structures presented here could be well utilised as synthetic reactors capable of combining enzyme and metal catalysis in a single system^35^, as shown by the successful spatial segregation of β-gal and FDG. Moreover, the UV-responsive functionality could play a dual role in such processes as nanomaterial synthesis, by enabling controlled mixing and simultaneously catalysing reactions between the mixed components or an orthogonal process external to the reactor^36,37^.

In summary, we have reported the engineering of light-responsive, nested vesicle reactors capable of responding to UV irradiation. User-controlled nanopore formation enables controlled mixing of small molecules between the two compartments. We have shown this through FDG release and hydrolysis by β-gal in bulk, as well as successful compartmentalisation and activity of the system in nested vesicle reactors. Future work will consider the encapsulation of enzyme cascades, separated within different internal lipid compartments, as well as the use of alternative chemical motifs to increase biocompatibility. Such structures should find use in the spatiotemporal control of reaction processes in diverse fields, from biocatalysis to on-site synthesis of therapeutics.

## Methods

### Production of nested vesicles by EPT

To prepare lipid films 10 mg of lipid was weighed out and dissolved in chloroform. This lipid solution was then gently mixed for 2 min, before evaporating the chloroform under a stream of N_2_(g) and storing the resultant film under vacuum overnight at room temperature. All films containing DC_89_PC were prepared under yellow light to avoid ambient photopolymerisation of the lipid during film preparation, and covered with foil during subsequent vesicle preparation.

Films were rehydrated with 0.75 mM fluorescein di-β-D-galactopyranoside 20 mM HEPES, 100 mM KCl, pH 7.4, at 50 °C, and freeze–thawed 6 times, flash–freezing the suspension in N_2_(l) each time before heating to 50 °C and vortexing for 2 min at 50 °C. The formed multilamellar vesicles were then extruded 21 times through a 200 nm polycarbonate filter at 50 °C to form a population of LUVs. The vesicles were then placed at 4 °C for 30 min in order to reverse any thermal activation of the vesicles before purification via SEC using a Sephadex G-50 column.

Nested vesicles were prepared using the EPT method, which allowed LUVs to be encapsulated in GUVs. This method uses water-in-oil emulsion droplets as templates around which lipid bilayers are assembled, with the lipid present in the oil phase making up the eventual GUVs. POPC lipid was dissolved in mineral oil (10 mg ml^-1^) by sonication for 30 min. The emulsion droplets, which become the internal compartments of the GUVs, contained purified LUVs with encapsulated FDG (prepared as described in S1.1 and S1.5), β-Gal (5 Uml^-1^) and sucrose (0.2 M), all in buffer (20 mM HEPES, 100 mM KCl, pH 7.4). a volume of 20 µL of this solution was added to 200 µL of the lipid-in-oil solution and vortexed for 30 s to create an emulsion. This was then layered above 250 µL of 0.2 M glucose in buffer in an Eppendorf, and centrifuged at 9000× *g* for 30 min to form a pellet of GUVs. The supernatant was removed and the pellet re-suspended in 250 µl 0.2 M glucose. A second centrifugation cycle was performed at 6000× *g* for 10 min, followed by removal of the supernatant and re-suspension of the GUVs in 250 µL 0.2 M glucose solution.

### Optical and fluorescence microscopy of nested vesicles

GUVs were transferred to a 1 mm deep circular polydimethylsiloxane well with a glass substrate coated with bovine serum albumin. The well was sealed with a coverslip, exposed to UV and imaged.

An inverted fluorescence microscope (Olympus IX-81) equipped with a fluorescence lamp (X-Cite 120, EXFO photonic solutions) and a CCD camera (Q-Imaging Retiga EXi fast) was used to image GUVs. A standard fluorescein isothiocyanate filter was used for fluorescence imaging, with 1 s exposure times. The lamp was turned off immediately after image acquisition to minimise photobleaching. Images were analysed using ImageJ, with vesicle boundaries detected using an inbuilt thresholding function, and total vesicle fluorescence normalised with respect to vesicle volume.

### Quantifying relative encapsulation efficiency in LUVs

In order to estimate the effect of increasing mol% DSPE-PEG2000 on encapsulation efficiency, fluorometry of 100 nm vesicles containing 0.5 mM calcein was undertaken. A Cary Eclipse Fluorometer (Agilent Technologies, USA) was used to record the fluorescence emission (*λ*_ex/em_ = 494/514 nm) of entrapped calcein in different vesicle compositions. Recordings were performed in 96-well plates (Thermo Scientific Nunc X50 Microplate Microfluor black) for 10 min on triplicate wells to obtain average fluorescence emission measurements for each composition. Relative encapsulation efficiencies were then calculated by normalising against the mean fluorescence for 0.5 mol% DSPE-PEG2000 vesicles, as this was the lowest PEG concentration that resulted in stable DPPC:DC_89_PC vesicles.

### Dynamic light scattering of LUV samples

All lipid vesicle sample sizes were verified via dynamic light scattering, using a Delsa™ Nano C Particle Analyser (Beckman Coulter, USA). 50 μl of vesicles were diluted in 350 μl of 20 mM HEPES, 100 mM KCl, pH 7.4 buffer or deionised water, and the size distribution of the particles measured (three runs, 75 measurements per run).

### Cryo TEM of LUVs

Bright-Field TEM was carried out on a JEOL 2100Plus (JEOL, Japan) using a minimum dose setup. Vesicles prepared in DI water at a 10 mg/ml concentration were irradiated for 10 min with UV-C before being prepared under cryo conditions to retain their structure using a Leica EM GP cryo plunge freezer. A volume of 5 µl of the sample was pipetted onto a holey carbon grid and the sample was allowed to settle on the grid for 30 s before blotting. The side where the sample was deposited was blotted twice for 1 s to remove any excess solution before rapid freezing in liquid ethane. The frozen sample was then transferred to the TEM under liquid nitrogen conditions.

### UV-vis spectroscopy of LUVs

All spectra were recorded on LUV samples diluted in 20 mM HEPES, 100 mM KCl, pH 7.4, buffer, using a NanoDrop 2000 (ThermoFisher, USA). Samples were recorded from 200–600 nm, and each spectrum was produced from the average of three drops, with three separate readings per drop.

### Spectroscopic monitoring of FDG release from LUVs

The fluorescence of all vesicle samples was recorded in 96-well plates, with FDG-derived fluorescein fluorescence emission recorded at *λ*_ex/em_ = 490/514 nm.

For irradiation experiments, sample fluorescence was recorded before irradiation in order to obtain background levels. Irradiation was then carried out, ensuring UV free samples were removed from the well plate at this point. After irradiation, fluorescence emission measurements were recorded every 5 min after irradiation for two hours to observe any changes in fluorescence. After two hours, 4 (v/v)% Triton X-100 was added to each solution to solubilise vesicles, which were mixed and left to fully solubilise for 30 min before recording the total solution fluorescence. Both DPPC:DC_89_PC:DSPE-PEG2000 vesicles and control vesicles lacking DC_89_PC were irradiated using this method.

### UV-C irradiation of vesicles

All fluorometry samples were irradiated in a 96-well plate whilst microscopy samples were imaged in fabricated PDMS wells. All irradiation was carried out with a UVG-11 compact UV lamp (254 nm shortwave UV emission, 230 V, 4 W) (UVP, USA). Irradiation occurred around 7.5 cm away from the sample in each case, and varied in time from 2.5–60 min irradiation. All experiments were performed at room temperature.

### Data availability

All relevant data are available from the authors upon reasonable request.

## Electronic supplementary material

Supplementary Information
