# Supplementary material for: Light-triggered enzymatic reactions in nested vesicle reactors
Source: Nat Commun. 2018 Mar 15;9:1093. doi: 10.1038/s41467-018-03491-7 (PMC5854585; doi:10.1038/s41467-018-03491-7)
Supplement: Supplementary file 1 — Supplementary Information [file 41467_2018_3491_MOESM1_ESM.pdf]

Light-triggered Enzymatic Reactions in Nested Vesicle Reactors

Hindley *et al.*

Supplementary Information

Supplementary Figures

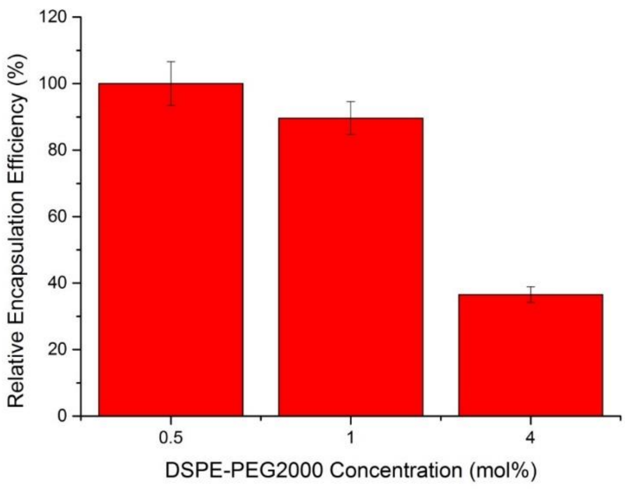

Supplementary Figure 1. Using calcein fluorescence spectroscopy to quantify the relative encapsulation efficiency of UV-responsive vesicles with increasing mol% of DSPE-PEG2000. Error bars represent 1 s.d. (n=3).

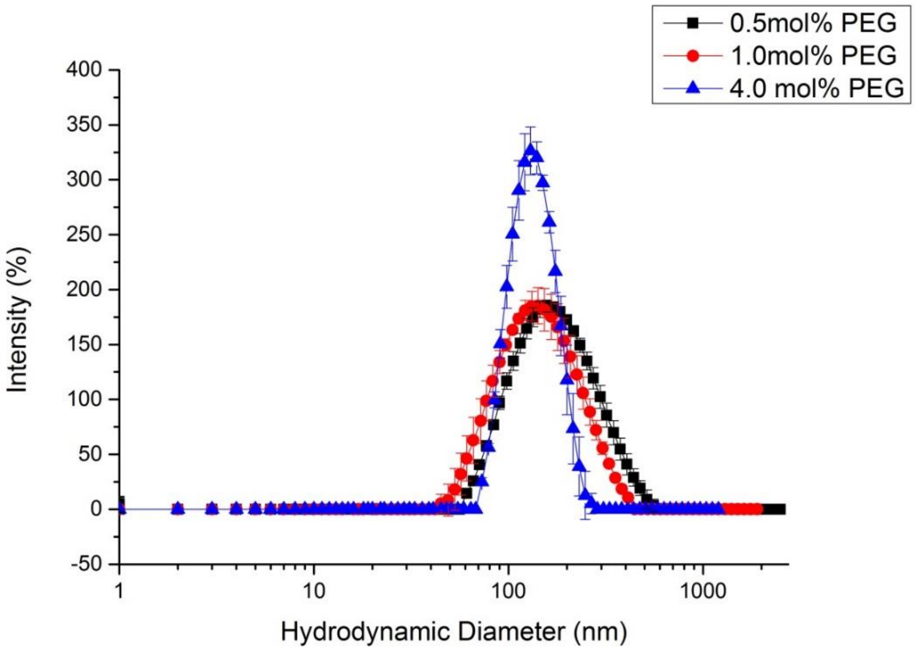

Supplementary Figure 2. Measuring the effect of increasing mol% of DSPE-PEG2000 on size stability of UV-responsive vesicles by Dynamic Light Scattering. Error bars represent 1 s.d. (n=3).

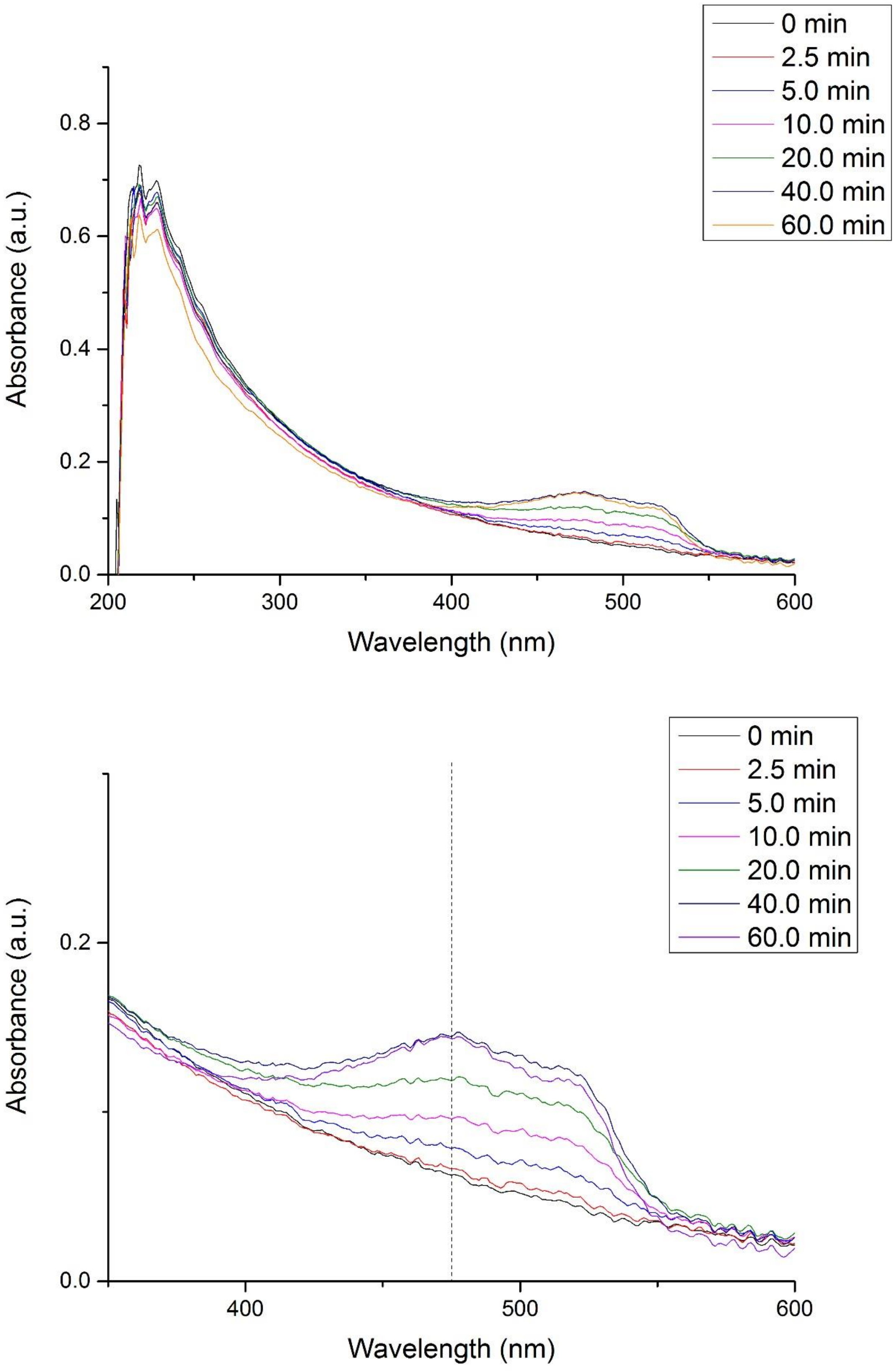

Supplementary Figure 3. Full UV-vis absorbance spectrum of DPPC:DC_{89}PC:DSPE-PEG2000 (79.5:20:0.5) UV-responsive vesicles with increasing UV-C irradiation, and zoom-in of the region of interest, with line indicating the wavelength used for normalisation of changes in the spectra.

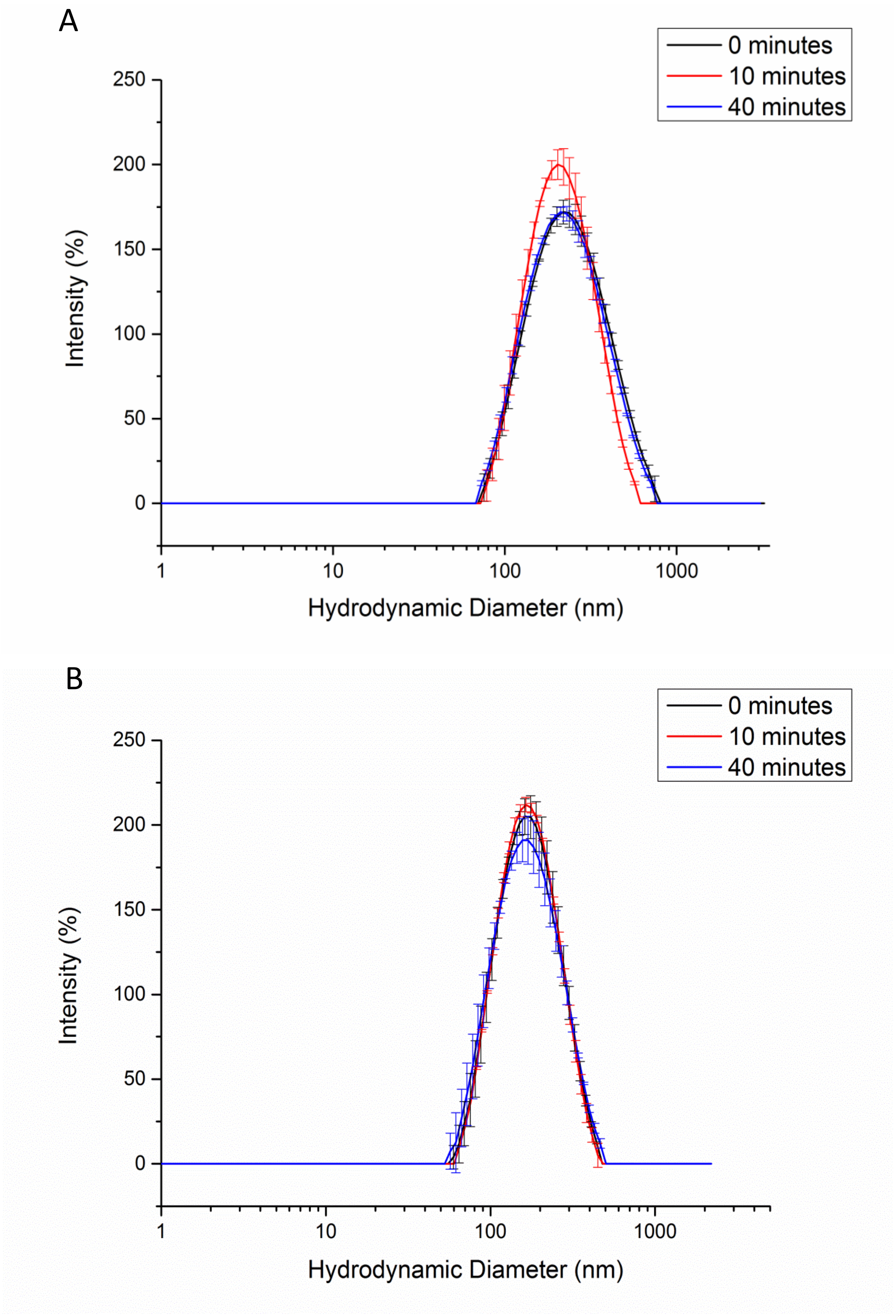

Supplementary Figure 4. Measuring the effect of UV-C irradiation on size stability of DPPC:DC_{89}PC:DSPE-PEG2000 (79.5:20:0.5) vesicles extruded through a 200 nm filter, suspended in **A)** 20 mM HEPES, 100 mM KCl, pH 7.4 and **B)** Deionized water. Error bars represent 1 s.d. (n=3).

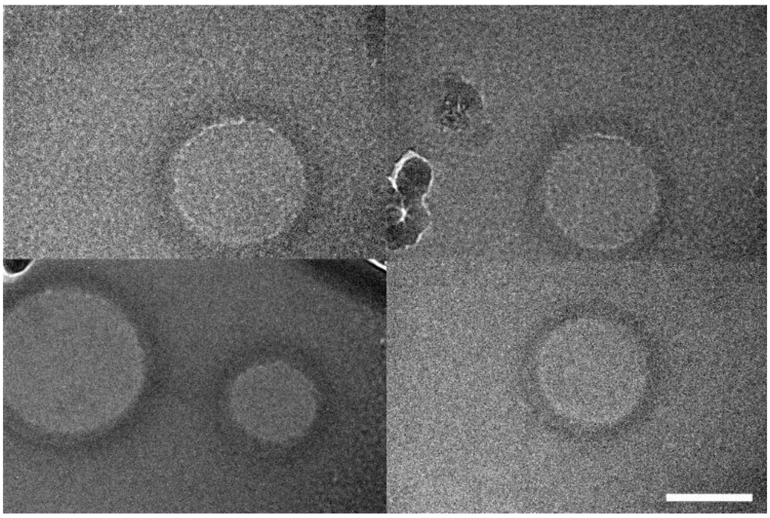

Supplementary Figure 5. Four example transmission electron micrographs (TEM) of DPPC:DC$_{89}$PC:DSPE-PEG2000 (79.5:20:0.5) UV-responsive vesicles extruded through a 200 nm filter. TEM samples were prepared under cryogenic conditions after 10 minutes irradiation with UV-C. Scale bar = 200 nm.

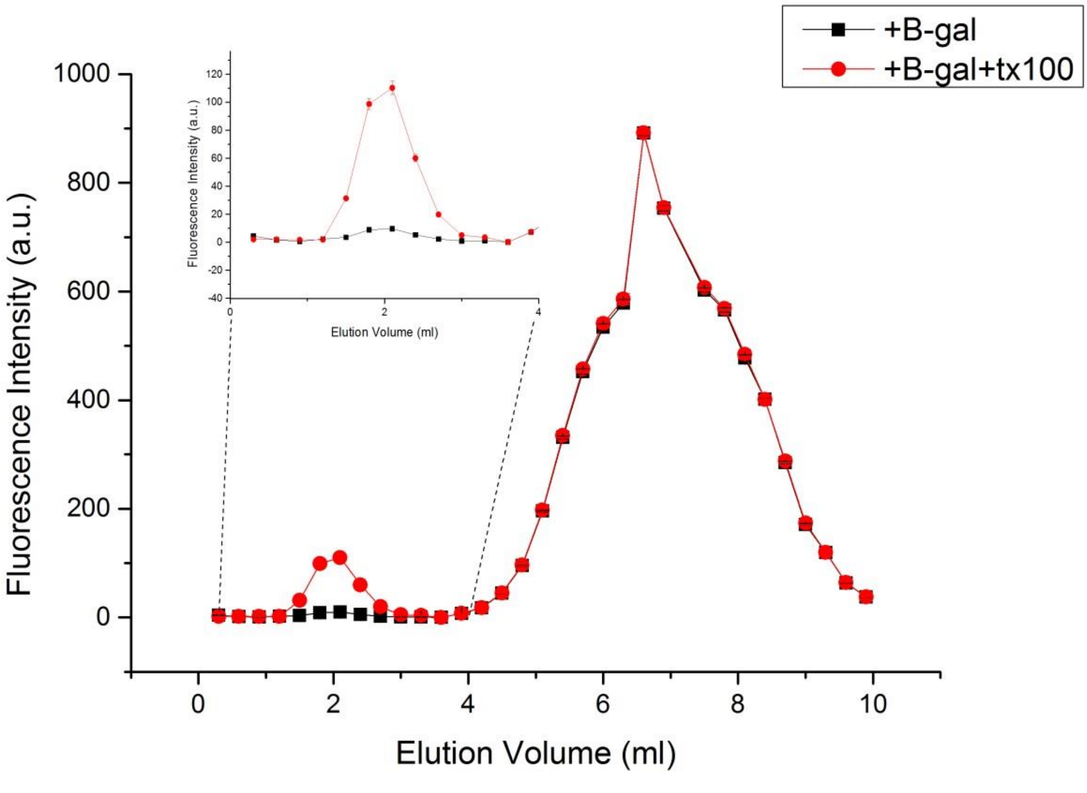

Supplementary Figure 6. Fluorometric elution profile of DPPC:DC$_{89}$PC:DSPE-PEG2000 (79.5:20:0.5) large-unilamellar vesicles and free-FDG, as measured by the fluorescence of hydrolysed FDG upon β-galactosidase addition. Note the clear resolution of two separate elution bands, with the first band consisting of UV-responsive vesicles, and the second free fluorescein. This can be

determined from the addition of the detergent Triton X-100, which lyses the vesicle membranes, allowing hydrolysis of encapsulated FDG to fluorescein.

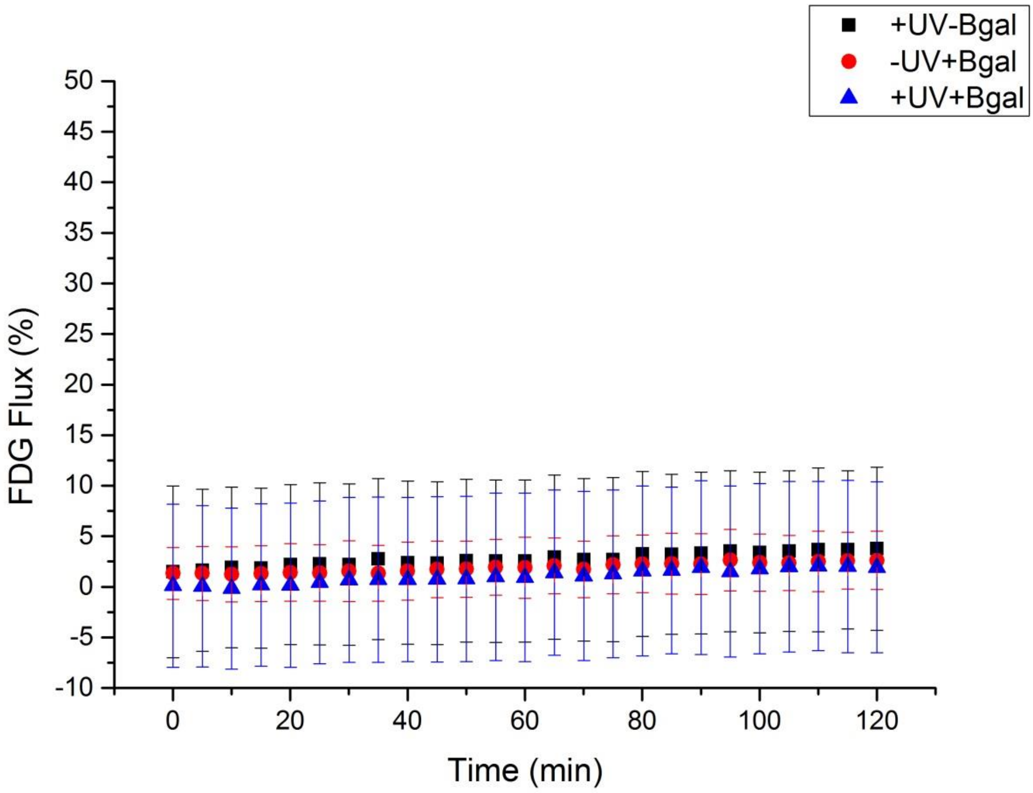

Supplementary Figure 7. UV-C irradiation of DPPC:DSPE-PEG2000 (99.5:0.5) vesicles does not result in the release of FDG, as measured by the fluorescence emission of fluorescein. Vesicles irradiated for 40 minutes before FDG release measured. Error bars represent 1 s.d. (n=3).
